# Splint: the efficacy of orthotic management in rest to prevent equinus in children with cerebral palsy, a randomised controlled trial

**DOI:** 10.1186/1471-2431-12-38

**Published:** 2012-03-26

**Authors:** Josina C Maas, Annet J Dallmeijer, Peter A Huijing, Janice E Brunstrom-Hernandez, Petra J van Kampen, Richard T Jaspers, Jules G Becher

**Affiliations:** 1 Department of Rehabilitation Medicine and the EGMO+ Institute for Health and Care Research and Research Institute MOVE, VU University Medical Center, De Boelelaan 1117, 1081 HV Amsterdam, The Netherlands; 2 Faculty of Human Movement Sciences and Research Institute MOVE, VU University, Van der Boechorststraat 7, 1081 BT Amsterdam, The Netherlands; 3 Departments of Neurology and Pediatrics, Washington University School of Medicine, St, Louis Children's Hospital, St. Louis, MO MO 63110, USA; 4 Medical Rehabilitation Center 'Groot Klimmendaal', Heijenoordseweg 5, 6813 GG Arnhem, The Netherlands

**Keywords:** Cerebral Palsy, Orthotic management in rest, Knee-ankle-foot orthoses, Ankle dorsiflexion range of motion, Prevention, Gastrocnemius muscle, Muscle morphology, Growth

## Abstract

**Background:**

Range of motion deficits of the lower extremity occur in about the half of the children with spastic cerebral palsy (CP). Over time, these impairments can cause joint deformities and deviations in the children's gait pattern, leading to limitations in moblity. Preventing a loss of range of motion is important in order to reduce secondary activity limitations and joint deformities. Sustained muscle stretch, imposed by orthotic management in rest, might be an effective method of preventing a decrease in range of motion. However, no controlled study has been performed.

**Methods:**

A single blind randomised controlled trial will be performed in 66 children with spastic CP, divided over three groups with each 22 participants. Two groups will be treated for 1 year with orthoses to prevent a decrease in range of motion in the ankle (either with static or dynamic knee-ankle-foot-orthoses) and a third group will be included as a control group and will receive usual care (physical therapy, manual stretching). Measurements will be performed at baseline and at 3, 6, 9 and 12 months after treatment allocation. The primary outcome measure will be ankle dorsiflexion at full knee extension, measured with a custom designed hand held dynamometer. Secondary outcome measures will be i) ankle and knee flexion during gait and ii) gross motor function. Furthermore, to gain more insight in the working mechanism of the orthotic management in rest, morphological parameters like achilles tendon length, muscle belly length, muscle fascicle length, muscle physiological cross sectional area length and fascicle pennation angle will be measured in a subgroup of 18 participants using a 3D imaging technique.

**Discussion:**

This randomised controlled trial will provide more insight into the efficacy of orthotic management in rest and the working mechanisms behind this treatment. The results of this study could lead to improved treatments.

**Trial Registration Number:**

Nederlands Trial Register NTR2091

## Background

### Cerebral palsy

*"Cerebral palsy (CP) describes a group of permanent disorders of the development of movement and posture, causing activity limitations that are attributed to non progressive disturbances that occurred in the developing fetal or infant brain. The motor disorders of cerebral palsy are often accompanied by disturbances of sensation, perception, cognition, communication, and behaviour, by epilepsy, and by secondary musculoskeletal problems" *[1]. Spastic CP is the most common form of CP (85%) [2]. Muscle spasticity is a clinical symptom characterized by a velocity dependent resistance to passive stretch or movement [3]. At present, in developed countries, about 2 live born children per 1000 have Cerebral Palsy [4,5].

Range of motion (ROM) deficits in one or more limb joints are present in many children with spastic cerebral palsy with about the half of the children having ROM deficits in the ankle, knee and hip [6]. In clinical practice, it is assumed that a reduced ROM in a joint is caused by a relative shortness of the muscle tendon complex compared to the length of the bone and/or by enhanced stiffness of the muscle tendon complex [7,8].

The *Gastrocnemius muscle *is often spastic in children with CP [9]. As the *Gastrocnemius muscle *has origin at the femur and his insertion at the calcaneus, this muscle is a major determinant of the ankle and knee ROM. The *Gastrocnemius muscle *was found to be shorter and stiffer in children with CP (having reduced ankle dorsiflexion) compared to typical developing children [10] and is expected to play a major role in the cause of limited ankle dorsiflexion ROM (measured at full knee extension). This ankle dorsiflexion ROM may lead to equinus deformities in the ankle [11]. Furthermore, a short and stiff *Gastrocnemius muscle *may lead to a gait pattern with increased ankle plantar flexion and increased knee flexion in midstance [12,13]. Compared to children with typical gait patterns, children with deviated gait patterns are impaired in mobility [9] and are metabolically less efficient and less resistant to fatigue during walking [14]. To prevent equinus contractures and less efficient gait patterns, it is important to treat and prevent impaired ankle dorsiflexion [9].

### Effectiveness of stretch

It is recommended not to use surgical interventions to improve gait (and thus not to treat impaired ankle dorsiflexion ROM by using surgical intervention) until gait is matured [9]. Based on joint immobilization studies of animals, it is well known that sustained muscle stretch stimulates an increase in muscle length by addition of sarcomeres in series [15-17]. In analogy with these results it is expected sustained muscle stretch as treatment of spastic calf muscles, will lengthen these muscles and in particular the gastrocnemius. However, a systematic review about the effectiveness of passive stretching shows that there is conflicting evidence on whether passive stretching can increase the ROM in a joint in children with CP [18]. Two types of stretching were investigated: 1) Manual stretching and 2) Sustained muscle stretch. Manual stretching was defined as *"holding the targeting joint to the available end ROM manually for a set amount of time, expressed as seconds, and then releasing it" *[18]. Sustained muscle stretch was defined as *"holding the targeting joint to the available end ROM by mechanical means such as standing tables or position equipment for an extended period, expressed as minutes up to 5-7 hours a day" *(a duration of 30 minutes stretching was the most commonly chosen in the analysed studies) [18]. In this review it was concluded that there appears to be only some indications that sustained muscle stretch is preferable to increase joint ROM in children with CP compared to manual stretching.

### Orthotic management in rest

Although sustained stretch is not an evidence based treatment, it is often applied by the use of night splints that are part of the general management of children with CP [9,19,20]. "Night splints" are used during night and/or during rest periods during the day. Therefore, we prefer to use the term "orthotic management in rest" instead of "night splinting". Regarding the muscle of interest in this study, the *Gastrocnemius muscle *of children with spastic CP, sustained stretch is applied by using knee-ankle-foot orthosis (KAFO). Static KAFOs (with ankle and knee angle fixed) as well as dynamic KAFOs (with ankle angle imposed by a spring allowing movement) are used. These orthoses hold the ankle joint at the maximal angle of dorsiflexion at full knee extension.

Using KAFOs in rest could be more effective compared to using KAFOs during active moments of the day. It might be presumed that a KAFO with fixed knee joints limits mobility, and therefore, will likely not be worn during active moments of the day. Other orthoses, like AFOs that are often used during active parts of the day, do not necessarily stretch the Gastrocnemius Muscle as the knee is allowed to flex. Knee flexion will occur during, for example, walking and sitting. To the best of our knowledge, Tardieu et al. [21] is the only study that evaluates the efficacy of orthotic management in rest in children with CP. It reports the effectiveness of orthotic treatment at night in two children, but these results are not confirmatory, due to the limited study design: 1) the number of treated subjects (2) was small, 2) there was no control group, and 3) the subject's ankle dorsiflexion was measured in knee flexion rather than extension which is more consistent with a measure of the *Soleus muscle *length instead of the *Gastrocnemius muscle*. Therefore, more research is needed to establish whether their conclusions were correct and whether the *Gastrocnemius muscle *will adapt in the same way to sustained stretch as the *Soleus muscle*. Despite the reported limited evidence in the literature, the efficacy of orthotic management in rest is probably considered as general knowledge. It is supposed that a KAFO prevents for reduced ankle dorsiflexion ROM when the KAFO is worn for 6 or more hours a day.

The major aim of this study is to obtain insight in the efficacy of orthotic management in rest to prevent a reduction in ankle dorsiflexion at full knee extension (clinical part of the study). Differences in the efficacy of static and dynamic KAFOs will be investigated and compared as well.

### Morphological properties

Recent literature shows that the morphological properties of muscles in children with CP differ from those in typically developing children [22-24]. For example, muscle belly length and muscle volume are smaller in children with CP compared to typically developing children [10,22,23]. A smaller fascicle length and smaller muscle thickness is found as well in children with CP [10,24]. Mostly, the *medial gastrocnemius muscle *was investigated. However, very little is known about the development of these morphological properties during growth both in typically developing children and children with CP [25], and the mechanisms underlying the decreasing ankle ROM during development in children with CP are unknown. Such insight is required to improve treatments for preventing reduced ankle ROM. Since the ROM of a joint is thought to be determined by the passive slack length (i.e. the smallest length at which any force is exerted) of the muscle tendon complex in relation to bone length and by the muscle tendon complex stiffness, improved knowledge of the changes in muscle morphology will likely provide insight into the etiology of reduced ROM. It is expected that the passive slack length of the muscle tendon complex will be affected by architectural variables, such as fascicle length (ℓ_(fasc)_), muscle belly length (ℓ_m_), physiological cross- sectional area (A_f_), angle between fascicle and aponeurosis (*γ_(fasc)_) *and tendon length (ℓ_t_) [26-29]. The muscle tendon complex stiffness is determined by the size and length of the muscle belly fibres and by the amount and arrangement of connective tissues (parallel elastic components) of the muscle tendon complex [30].

**Figure 1:**
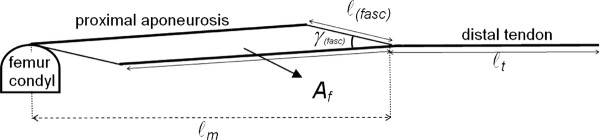
**Schematic overview of the different architectural parameters that determine the length of the muscle tendon complex of the *medial Gastrocnemius muscle***. Symbols are explained in the text.

The KAFO treatments tested in this study are assumed to prevent the development of a reduced ankle dorsiflexion at full knee extension by increasing the slack length of the *Gastrocnemius muscle *muscle tendon complex and by reducing the muscle tendon complex stiffness. However, even if effective, the question remains whether the muscle tendon complex slack length increases due to muscle architectural changes like increased fascicle length or due to the amount and arrangement of connective tissues of the muscle tendon complex or both (see Figure 1 for an overview of the different architectural parameters that determine the length of the *medial Gastrocnemius muscle*.

Therefore, the secondary aim of this study is to evaluate how changes in ankle dorsiflexion are related to morphological changes in the *medial Gastrocnemius muscle*.

### Hypotheses

#### Clinical part

We hypothesize that children with Cerebral Palsy (CP) who are treated with a knee-ankle-foot orthosis (KAFO) will show a smaller decline in (or even increase in) ankle range of motion (ROM) into dorsiflexion compared to children not being treated with a KAFO. In addition, we anticipate that children who are treated with a static KAFO will show less of a response (i.e. they will have a larger decline or a smaller increase in ankle ROM into dorsiflexion) when compared to children being treated with a dynamic KAFO.

We expect that for children with CP, treatment with either KAFO will have a less negative change in gait or even a positive change in gait pattern. A positive change refers to less ankle plantar flexion and less knee flexion in midstance, compared to no KAFO treatment. The effects are expected to be more positive in children being treated with the dynamic KAFO compared to the children being treated with the static KAFO.

The level of mobility (GMFM-score) of children who are treated with either KAFO is expected to show a smaller decline or increase compared to children who are not treated with a KAFO. As above, the effects are expected to be larger in children being treated with the dynamic KAFO rather than in children being treated with the static KAFO

#### Morphological part

In this study, it is hypothesized that the tendon length, muscle belly length and fascicle length increase and the muscle tendon complex stiffness decreases due to exposure of the *medial Gastrocnemius muscle *to sustained stretch.

## Methods

This study is approved by the Medical Ethics Committee of the VU University Medical Center and by the Institutional Review Board of the Washington University in Saint Louis.

### Participants

#### Criteria

The specific inclusion and exclusion criteria are shown in Table 1. Briefly, participants are children with spastic CP having been treated for reduced ankle dorsiflexion in the past, but not needed to be treated at the moment they were included into this study. Children are excluded from the study if they have a history of surgery of the *Gastrocnemius muscle *and/or *Soleus muscle *and/or selective dorsal rhizotomy, if they have severe enough morbidity or mobility limitations that prevent them from walking far enough to complete a gait analysis, or if they are being treated with intrathecal baclofen therapy (i.e. they have a current, active pump).

**Table 1:** Inclusion and exclusion criteria

Inclusion criteria	Exclusion criteria
Children must have: 1. a clinical diagnosis of unilateral or bilateral spastic CP 2. an age between 4-12 years old 3. at least 0° ankle dorsiflexion with extended knee (physical examination) 4. a GMFCS class I, II or III 5. has been treated for reduced ankle dorsiflexion (< 5° dorsiflexion) before the start of the study by: a. and/or serial casting at least 3 months ago b. and/or botulinum toxin A injections in the *Gastrocnemius *and/or *Soleus muscle *at least 6 months ago c. orthotic management in rest with a knee-ankle-foot orthosis to prevent for decreasing ankle dorsiflexion 6. a stable social family situation	Children must not: 1. have had surgery of the *Gastrocnemius *and/or *Soleus muscle* 2. have had Selective Dorsal Rhizotomy 3. have had Intrathecal Baclofen therapy 4. have had Botulinum toxin A treatment in the lower extremity less than 6 months ago 5. have had casting of the lower extremity less than 3 months ago 6. have knee contractures (less than 0° knee extension) 7. have more than 20° ankle dorsiflexion at full knee extension 8. have behavioural problems (like severe mental retardation) 9. have significant sleeping problems 10. be institutionalised 11. be suffering from co-morbidity interfering with mobility that prevents them from walking adequate distance. 12. have problems with understanding either the Dutch (for subjects in the Netherlands) or English language

#### Sample size calculation

Expecting a 5 degree change in ankle ROM (assumed as clinically relevant), with a standard deviation of 4.5 degrees, a significance level (α) of 0.05 that is corrected for comparisons between three groups using a Bonferroni correction (α = 0.0167), and a power level of 80%, 13 children in each group will be sufficient. The calculation takes five repeated measurements with a correlation coefficient of ρ = 0.7 into account. In this study, 66 participants (22 in each group) will be recruited to allow drop outs.

#### Recruitment procedure

Subjects will be recruited from three centers: 1) the VU University medical center in the Netherlands (N = 18), 2) "Rehabilitation Medical Center (RMC) Groot Klimmendaal" in the Netherlands (N = 18) and 3) the Pediatric Neurology Cerebral Palsy Center at Washington University School of Medicine and St. Louis Children's Hospital in the USA (N = 30). Eligible subjects will be identified by the physicians during clinical sessions or from review of patient's charts. The recruited children and their parents will receive a letter about procedures and content of the study, as well as an informed consent form. The potential subjects and their caregivers will be informed by the site investigators and physicians. Both parents/guardians and children being 12 years old are asked to sign and return the informed consent to agree on voluntary participation in the study.

### Setting & design

A single blind randomized controlled trial will be performed at the three above mentioned centers. The participants will be assigned into 3 different groups. In addition to their regular treatment, two groups will be treated with a dynamic or static KAFO for one year to prevent for a reduction of ankle dorsiflexion at full knee extension and one group will be included as a control group without additional intervention. The morphological measurements will be performed only at Dutch participants at the VU University medical center.

Measurements will be performed at baseline and at 3, 6, 9 and 12 months after treatment allocation. In combination with those measurements, participants of the experimental groups will have a meeting with the orthotist and physician to check for complications with the KAFO.

The assessor and analyser are blinded for treatment allocation. The trial will be performed between January 2010 and December 2012.

### Intervention/comparisons

Patients of the control group will receive their usual care which may include ankle-foot-orthoses (AFOs) that are worn during the day (for standing and walking), oral baclofen therapy or other tone-reducing medications, strength training, stretching exercises, physical therapy, occupational therapy etc. Changes in usual care during the study will be monitored using questionnaires. Children will drop out of the study if they need surgical treatment (orthopaedic and neurosurgical procedures affecting muscle tone and length), botulinum toxin A injections in the lower extremity or serial casting treatments in the lower extremity.

In addition to their usual care, patients of the experimental groups will be treated for one year with a static KAFO or with a dynamic KAFO using an Ultraflex^® ^ankle power unit. Children will be asked to wear the KAFO at least 6 hours per night. They will be allowed to remove the KAFO during the night when the child is seriously uncomfortable after wearing the KAFO for at least 20 minutes or when the child wakes up at night with complaints concerning the KAFO. When children do not wear the KAFO for 6 hours per night, parents will be asked to increase wearing time by asking the child to wear the KAFO during rest activities in day time. In case of unilateral treatment, patients will sleep one night with and one night without a KAFO. In case of bilateral treatment, patients will wear a KAFO alternating on the right and left side each night.

#### Manufacturing the KAFO

**Figure 2:**
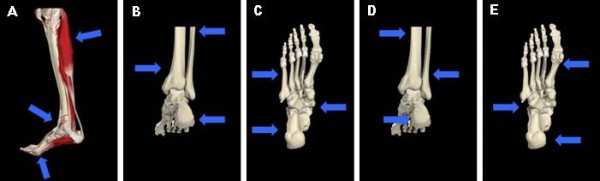
**Three point's pressure for correction of deformity**. (**a**)The equines correction will be performed by exerting force on the dorsal side of the lower leg (just below the knee), on the instep of the foot and under the ball of the foot. (**b**)The valgus correction of the calcaneus will be performed by a exerting a force laterally on the heel/calcaneus, laterally on the middle of the lower leg and medially on the lower leg, just above the medial malleolus. (**c**) The forefoot abduction correction will be performed by exerting a medial stabilization force calcaneus and talus and a lateral force on the calcaneus en the fifth os metatarsi. (**d**) The varus correction of the calcaneus will be performed by exerting force on the medial part of the calcaneus, medially on the middle of the lower leg and laterally on the lower leg, just above the lateral malleolus. (**e**)The forefoot adduction will be performed by exerting force on the tuberositas of the fifth os metatarsi, by exerting force laterally on the calcaneus and laterally on the first metatarsal phalangeal joint.

The KAFO will be custom made by certified orthotists using polyethylene or polypropylene and foam (for a covered inside). Two transverse bars (polyethylene or polypropylene) above and below the knee will be used to reinforce and stiffen the KAFO. Bandages of nylon Velcro straps will be placed at three locations: 1) as high as possible on the thigh, 2) directly above the patella and 3) directly below the patella. A circular foot fixation, made of leather or soft polyethylene, will be used for foot fixation. This circular foot fixation will be closed with two velcro straps. One strap overlaps the patient's most convex part of the ankle and one strap will overlap the patient's foot proximal of the caput ossis metatarsal I and V. Deformity of the patient's foot will be corrected by the use of an internal three point pressure system (see Figure 2).

#### Static KAFO specification

The static KAFO will provide a fixed knee extension of 0° and a fixed ankle dorsiflexion angle of 0°.

#### Dynamic KAFO specification

**Figure 3:**
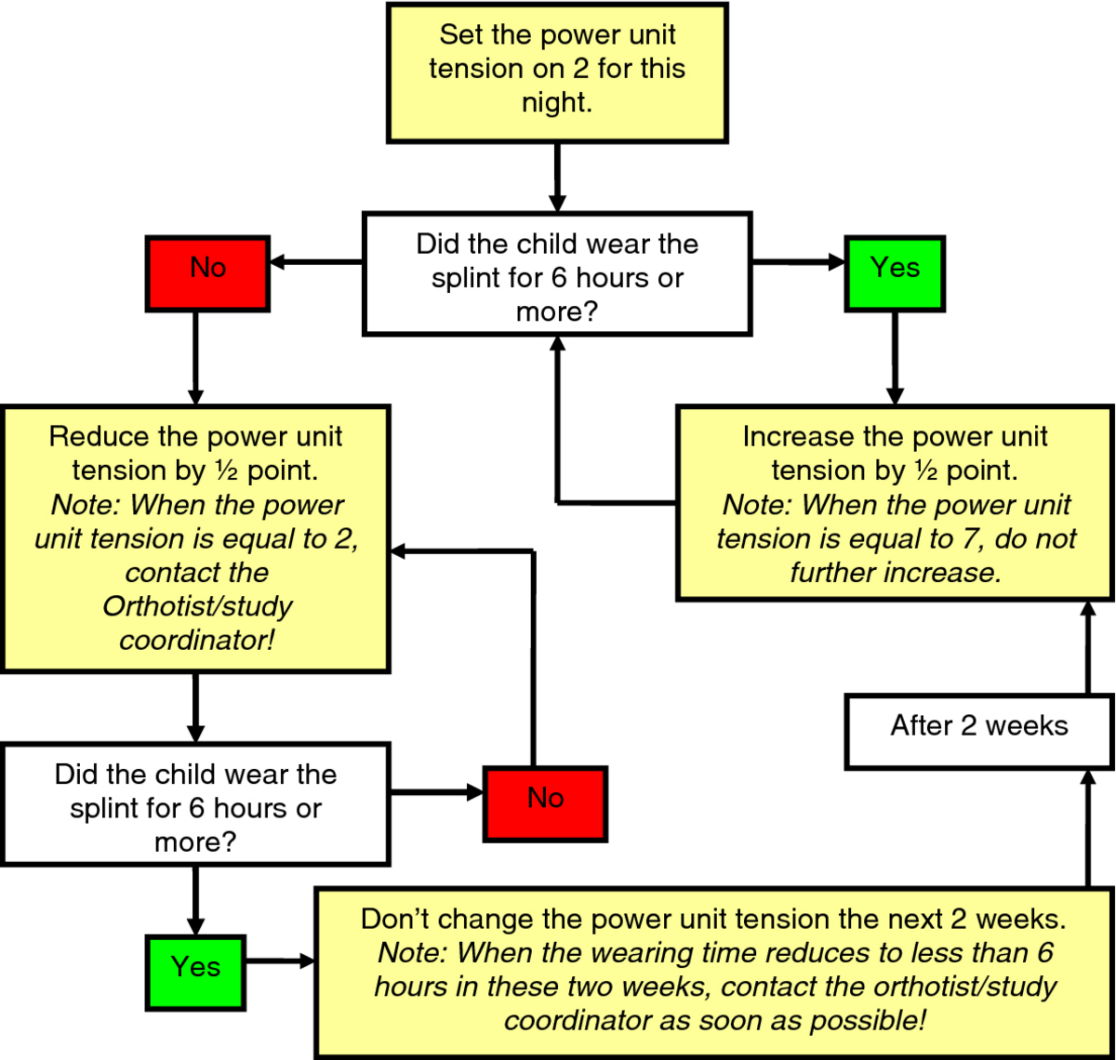
**Manual for dynamic splint settings**.

The dynamic KAFO will also have a fixed knee extension of 0°, but will use an ultraflex^® ^ankle power unit (Ultraflex Systems, Pottstown, PA, USA). The force of the power unit that provides variable ankle dorsiflexion angles will be set according to the prescription shown in Figure 3.

### Outcome measures

#### Primary outcome

**Figure 4:**
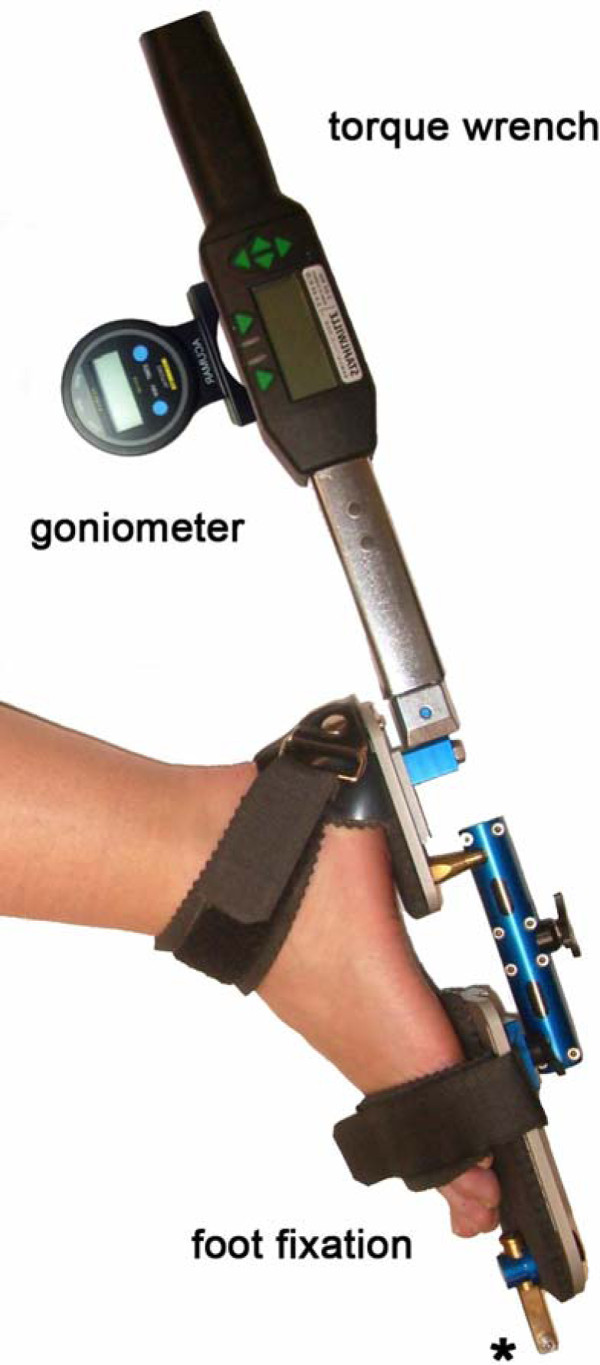
**Photographic illustration of the hand held dynamometer**. The hand held dynamometer consists of an adjustable foot fixation, a torque wrench and a goniometer. The foot fixation has parts supporting the forefoot and calcaneus. These parts are connected by a rod, allowing independent adjustments in rotation and abduction/adduction. The forefoot part is equipped with a fixation point to the table when needed (*).

To measure the maximal ankle dorsiflexion angle at full knee extension, a Single Digital Inclinometer (Model ACU001, Acumar, Lafayette, IN, USA) will be used. This goniometer is attached to a torque wrench (Sensotork 713/6, Stahlwille, Germany). The goniometer-torque wrench combination is attached to an adjustable foot fixation. The foot fixation is constructed with a forefoot part and a calcaneal part. The two parts can be adjusted in rotation and in distance with respect to each other. With the adjustment in rotation, adjustments for fore foot adduction and supination can be made to stabilize instable valgus foot deformity. With the adjustment in distance, foot sizes can be accommodated from 150 to 240 mm. The calcaneal part has a heel support (width: 45 mm) and a point to attach the torque wrench. Both parts are equipped with Velcro straps for foot fixation [31]. Figure 4 shows a photograph of the measurement device attached to the foot. The ankle dorsiflexion angle will be measured as the angle between the footplate of the foot fixation and the tibia (*γ_(f-t)_*).

The children will be asked to lie prone on a bench, with both feet overhanging the edge. The lower leg will lie in such a way that the fibula head and the lateral malleolus of the fibula are on the same height. The foot will be firmly attached to the adjustable foot plate for fixation. The ankle will be plantar flexed by the researcher, applying an external plantar flexion moment of 4 Nm, as measured using the toque wrench. The corresponding *γ_(f-t) _*is measured (further described as the 4 Nm plantar flexion angle). Subsequently, this procedure is repeated for 1 Nm plantar flexion and, 0 Nm, 1 Nm dorsiflexion, 4 Nm dorsiflexion and 6 Nm dorsiflexion. All measurements will be repeated six times and each moment will be exerted for five seconds. The *γ_(f-t) _*will be read out from the inclinometer simultaneously at the end of these 5 seconds at the target ankle joint moment. Positive values refer to an external dorsal flexion moment (Nm) of the dynamometer and dorsal flexion angle (°) of the ankle joint. There will be five seconds rest between each repetition and two minutes rest between each condition. The conditions will always be applied in the described order.

Children have to relax their muscles and will be asked to lie quietly during the measurements. Muscle activity will be checked using the electromyography (EMG) signals of *Tibialis anterior muscle *and *lateral Gastrocnemius Muscle*. The maximal voluntary muscle contraction (MVC) will be recorded before the measurements. The EMG signal will be A-D converted at 1000 Hz. After sampling, the signal will be high-pass filtered at 20 Hz to remove movement artefacts. Then, the signal will be normalized with respect to the MVC-value and filtered low pass at 5 Hz. EMG signals have to remain below 10% MVC during the angle and moment measurements to ensure muscle relaxation. Skin preparation and electrode placement of EMG will be carried out according to SENIAM guidelines [32].

**Figure 5:**
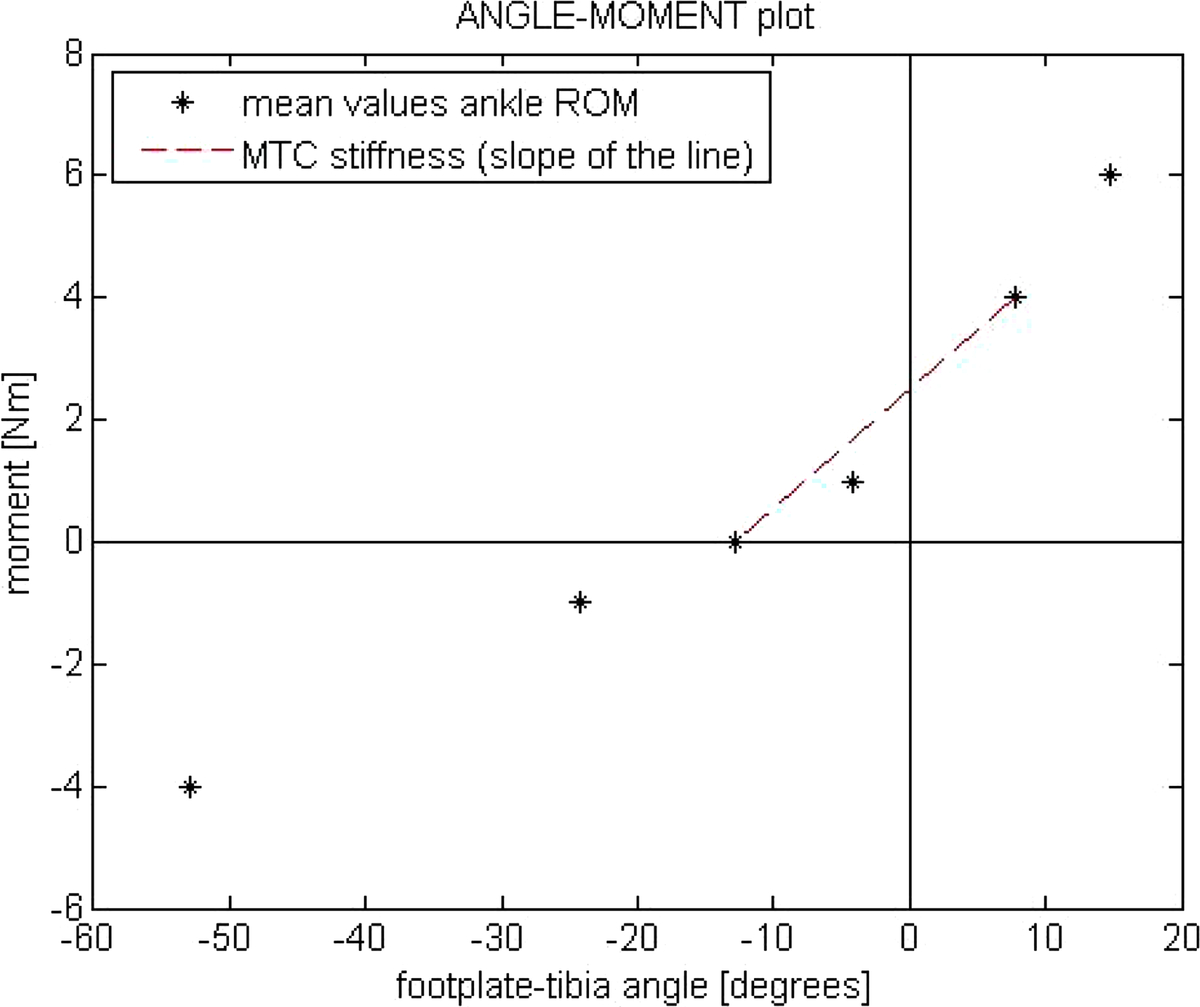
**Ankle-moment plots**. This figure will be created from the values measured with the hand held dynamometer. The dotted line will be used to calculate the muscle tendon complex (MTC) stiffness by calculating the slope of that line.

The mean of the first 5 measurements for each condition in which the EMG signal remained below 10% MVC will be used for further analysis. The results will be used to create angle-moment plots in which, for example, the muscle tendon complex stiffness can be determined by calculating the slope of the line between the 0 Nm and 4 Nm (see Figure 5). A change in ankle dorsiflexion ROM will be investigated by analysing the *γ_(f-t) _*measured with 4 Nm dorsiflexion.

In case of potential bilateral treatment, the full procedure will only be performed on the participant's most involved leg. For the other leg, only the 4 Nm condition without EMG measurement will be performed to check for exit criteria (see withdrawal paragraph below). In case of potential unilateral treatment, the primary outcome measure will only be measured for the participant's potentially treated leg.

#### Secondary outcome - gait analysis

Sagittal and frontal video-recordings of the patient's gait pattern will be made at 50 Hz. The subjects will walk 5 times barefoot and 5 times with shoes and AFO if applicable, along a 10 m walkway at self-selected comfortable speed. Walking speed will be calculated from the time to complete a part of the track (5 meters, measured with infra red detectors or with a stopwatch, depending on measurement location). For follow up measurements, the patient will be requested to walk at baseline walking speed (within a range of ± 5%). Video recordings of the involved leg(s) will be taken in the sagittal and frontal plane. Three representative steps will be chosen for the assessment of the knee angle in midstance, the minimum knee angle in stance (between midstance and second bipedal phase of foot contact) and the ankle flexion in midstance. For the video analysis, a custom-made software package will be used (the Moxie Viewer^®^, VU University Medical Center, Amsterdam, the Netherlands, http://www.smalll.nl), and a software tool, allowing on screen video measurements of sagittal lower extremity joint angles [33]. For all participants, the gait related outcome measures will only be measured in the potentially treated legs.

#### Secondary outcome - mobility

The level of mobility will be quantified using the Gross Motor Function Measure 66 Item Set (GMFM-66 IS) [34] by a certified assessor. GMFM-66 IS scores will be calculated with the corresponding software (Gross Motor Ability Estimator version 1.0) that calculates scores on an interval scale.

#### Patient characteristics

Patient characteristics will be recorded using an intake form and will include age, gender, race, ethnicity, weight, length, localisation of CP (uni- or bilateral) and Gross Motor Function Classification System (GMFCS) [35] class. To asses problematic behaviour of the child, the strength and difficulties questionnaire (SDQ) [36] will be filled in by the parents. In addition, questions will also be asked about the children's sport activities, current therapies and other treatments, as well as preference sleeping positions.

#### Physical examination

Physical examination will be performed by the assessor to evaluate the physical characteristics of the patient. Variables to be measured are: 1) Position of the foot in standing, 2) transmalleolar axis position [37], 3) gait pattern classification according to Rodda [38] and Becher [39], 4) ROM of the ankle and knee joints, 5) spasticity, by measuring the angle of catch (AOC) [40] of the ankle and knee, 5) selective motor control, using the Selective Control Assessment of the Lower Extremity (SCALE) [41] and 6) lower leg length. For all participants, the physical examination related outcome measures will only be measured in the potentially treated legs.

#### Protocol adherence

Web based diaries will be used to record the protocol adherence and will be collected by a research assistant. These diaries will be filled in during the fourth week of each month and include questions regarding KAFO use, KAFO-related complaints, sleeping problems, the use of an ankle-foot orthosis (AFO) as well as questions regarding stretching exercises performed over the last month. Problems with KAFO use experienced by patient and/or parents will be monitored by specific diary questions. The research assistant will call the participants at least once a month to check if there are any problems with the KAFO or motor function of the participant. Reported problems will be solved as soon as possible.

Furthermore, to check the reliability of diary reported KAFO wearing time, wearing time of the splints (for a subgroup of 10 children, recruited at VU University Medical Center) will be determined on the basis of KAFO temperature measured using a TidBit temperature datalogger (UTBI-001, Onset Computer Corporation, Bourne, MA). The KAFO temperature will increase due to body heat when the KAFO is worn. A sample of KAFO temperature data will be recorded each 15 minutes during the treatment period. Parents and children are not informed about the purpose of this measurement.

#### Other

To get an indication of the sustained muscle stretch that is applied by the KAFOs, two measurements will be added. 1) The ankle moment at a *γ_(f-t) _of *0° to simulate the static KAFO condition. This condition will be performed before the handheld dynamometer protocol. 2) The ankle dorsiflexion angle that could be imposed by the dynamic KAFO will be estimated during consultation hours by the physician using a goniometer.

### Morphological part

**Figure 6:**
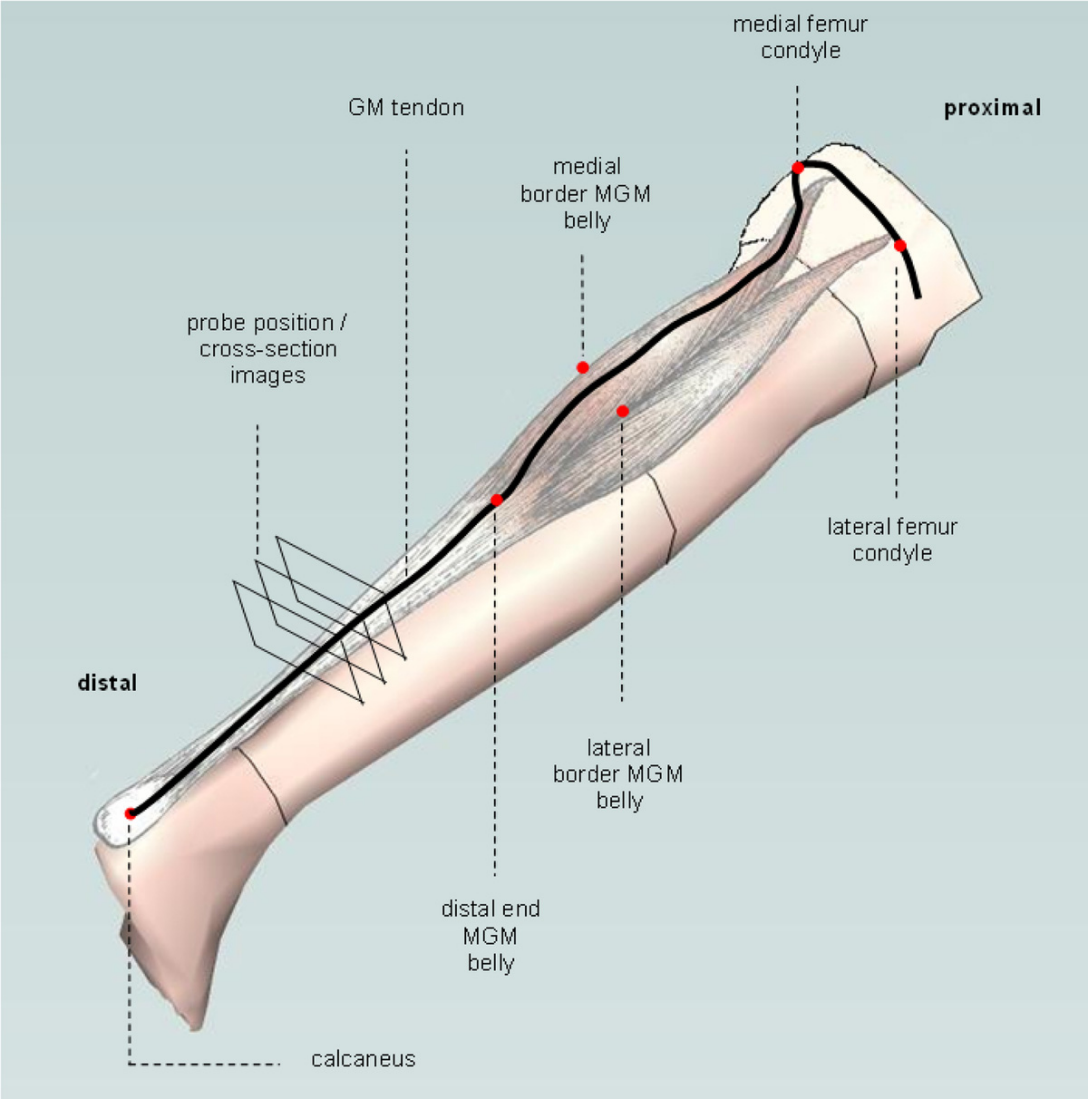
**Path of the ultrasound probe during scanning the *medial Gastrocnemius muscle *(MGM)**. The probe follows the path over the black line. It starts proximal, with the probe perpendicular to the path. First, the probe will be guided from lateral to medial over the respectively lateral and medial condyle of the femur. Then the probe will be rotated and moved to distal between the medial and lateral border of *medial Gastrocnemius muscle *belly towards the distal end of the muscle belly, the *Gastrocnemus muscle *(GM) tendon and the calcaneus.

To determine muscle morphology related variables, 3D-ultrasound imaging will be performed on the *medial Gastrocnemius muscle*. This muscle, covered with an ultrasound gel, will be scanned along it's length (making multiple transverse cross-section images, see Figure 6) using a 5-cm linear array probe (12,5 MHz) of a B-mode ultrasound device (Technos MPX, ESAOTE, Italy). Two sets of recordings of the *medial Gastrocnemius muscle *will be made for each session. During 3D-ultrasound measurements, the position of the probe with a cluster marker is recorded using an active 3D marker motion analysis system (Optotrak, Northern Digital, Waterloo, Canada). In addition, 6 anatomical landmarks (lateral malleolus, medial malleolus, medial femur condyle, lateral femur condyle, medial femur epicondyle and lateral femur epicondyle) are recorded before each experiment to gain an anatomical frame of reference for post experimental 3D image reconstruction. In a prone position, the children are lying quietly on a bench. Using the ankle dynamometer, the ankle is fixed at *γ_(f-t) _*corresponding to 0, 1 and 4 Nm net dorsal flexion moment. Muscle activity is checked using EMG during the ultra sound measurements as described above in the primary outcome section.

**Figure 7:**
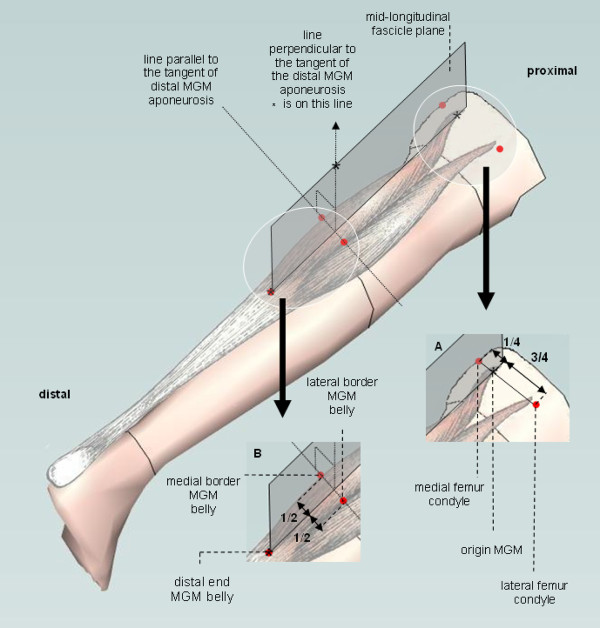
**The orientation of the mid-longitudinal fascicle plane**. Three orientation items (*) were used to define the mid-longitudinal fascicle plane of the *medial Gastrocnemius muscle *(MGM) (shaded plane and inset): 1) The estimate of the origin of the *medial Gastrocnemius muscle *(at 1/4^th ^of the line from medial to lateral condyle of the femur, see inset **A**) 2) the most distal muscle belly end, and 3) a point on the line perpendicular to tangent to the distal aponeurosis in the transversal plane. The direction of the tangent is determined in the distal part of the *medial Gastrocnemius muscle *belly exactly in between the *medial Gastrocnemius muscle *borders (see inset **B**).

The ultra sound images will be converted into a voxel array and 3D-reconstructions will be calculated using a custom made program in MATLAB software according to the method that was described by Bénard e.a.[42]. Measurements are performed in the mid-longitudinal fascicle plane of the *medial Gastrocnemius muscle*, being perpendicular to (the tangent of) the distal aponeurosis of the *medial Gastrocnemius muscle*, selected from the voxel array (see Figure 7). The use of the correct plane is essential for minimizing measurement errors of fascicle length, fascicle angle and muscle thickness [43]. Measurements are performed five times because this number of repetitions has been shown to yield reliable results [43].

The following variables will be measured: tendon length (ℓ_t_,) muscle length (ℓ_m_), fascicle length (ℓ_(fasc)_), muscle thickness (ℓ_(m th)_) and fascicle angles with the aponeuroses (*γ_(fasc)_)*.

**Figure 8:**
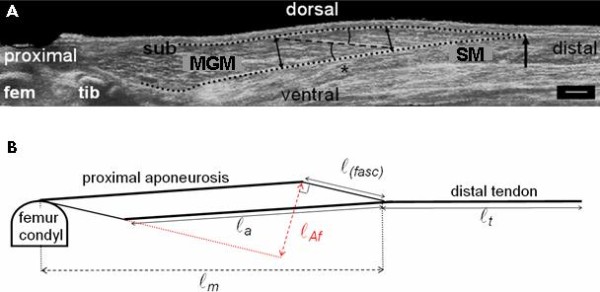
**Measurement and calculation of muscle geometry of *medial Gastrocnemius muscle *within its mid-longitudinal fascicle plane**. (**A**) The mid-longitudinal fascicle plane, determined with 3D ultrasound. The *medial Gastrocnemius muscle *(MGM) is covered by the subcutis (SUB) and supported by *Soleus muscle *(SM). Parts of both femur (fem) and tibia (tib) are shown. The black dotted lines define the outline of the muscle. The most distal muscle belly end is indicated by a black arrow. The length of the target fascicle (ℓ_(fasc)_) (dashed black line), centred at 2/3^rd ^(*) of muscle belly length (from the origin) is measured. Muscle thickness (ℓ_(m th)_) was calculated as the distance between the proximal and the distal aponeurosis at the proximal end of the target fascicle (left black double arrow). The fascicle-aponeurosis angle (*γ_(fasc)_) *was calculated as the mean of the angles of the fascicle with the proximal and distal aponeurosis (black arcs). Scale bar depicts 1 cm. (**B**) Schematic overview of morphological muscle parameters in the mid-longitudinal fascicle plane of the *medial Gastrocnemius muscle*. The length of the aponeurosis (ℓ_a_) and the length of the physiological cross-sectional area (ℓ_Af_) will be calculated from this model. The muscle belly length (ℓ_m_) will be measured from the origin at the femur condyle to the distal end of the muscle belly. The tendon length (ℓ_t_) will be measured from the distal muscle belly end to the insertion at the calcaneus.

Using trigonometry, the following variables will be calculated: length of *medial Gastrocnemius muscle *intramuscular distal (i.e. deep) and proximal (i.e. superficial aponeuroses) (ℓ_a_) and length component of the physiological cross-section (ℓA_f_, the added perpendicular diameters of fascicles within the mid-longitudinal fascicle plane, Figure 8).

In case of potential bilateral treatment, the morphological outcome measures will only be measured on the participant's most involved leg. In case of potential unilateral treatment, the morphological outcome measure will only be measured at the participant's potentially treated leg.

### Exit criteria

#### Withdrawal

The investigator and/or clinician can decide to withdraw a subject from the study for urgent medical reasons. First, they have an ankle dorsiflexion angle with an extended knee, measured as the angle between tibia and footplate (*γ_(f-t)_)*, of 10° plantar flexion or more when an external ankle dorsiflexion moment of 4 Nm is applied. In such a case, the assessor will refer the child to the clinician who will decide whether the reduction in ROM has to be treated or not (note that the net ankle dorsiflexion moment of 4 Nm applied by the assessors is lower than is typically applied in a clinical setting). These children will not undergo follow-up measurements as they will receive other treatment for impaired ROM. Second, children have irresolvable problems with KAFO use (pain, pressure sores, sleeping problems). These subjects will be asked to undergo measurements after withdrawal and will be included in the analyses. Third, children can decide to withdraw at any time for any reason. These children will be asked to undergo measurements after withdrawal as well and will also be included in the analyses.

#### Premature termination of the study

The effects of orthotic management in rest on ankle dorsiflexion at full knee extension will be evaluated as soon as measurements have been performed on 30 children regarding follow-up measurement of 6 months. If the children in the control group show significantly larger *γ_(f-t) _*reduction compared to the other groups, this group will also be treated with a knee-ankle-foot orthosis. If the knee-ankle-foot orthoses groups (static and/or dynamic) show significantly larger reductions in *γ_(f-t) _*than the control group, the study will be terminated.

### Randomization

Randomisation will be performed by block randomisation of 3, 6 or 9 subjects, with pre-stratification by center. A member of the project team (AJD) not being involved in the recruitment/inclusion procedure of the subjects and not being involved in measurements will randomly generate an allocation sequence before the start of the trial to perform the randomisation. The order of allocation of treatment will be noted by AJD and kept in numbered sealed envelopes. After checking the inclusion and exclusion criteria by a physician and after receiving informed consent of the participant's parents, treatment allocation will be established by the research assistant after opening the numbered envelope. Subjects will be informed about their allocation after performing their baseline measurement.

### Blinding

The researchers performing the measurements and analysing the data will be blinded with respect to the treatment allocation. The children and their parents will be instructed to give no information about their treatment to the assessors. Blinding will be evaluated at the end of the study by asking the researchers the question: "In which group is the subject allocated and do you know this for sure, or is this a guess?"

### Statistics

Statistical analysis will be performed according to an "intention to treat" principle. All relevant subject characteristics, such as age, body weight and length, gender, clinical diagnosis, will be described by their mean value and standard deviation, or percentages. Differences between groups at baseline will be tested using linear regression techniques or Chi-square statistics. The effect of the intervention will be analysed using a multi-level analysis, with the primary and secondary outcome measures as dependent variables and treatment group and time as independent variables. To test for any differences in the changes of variables between groups, a treatment*time interaction is included as independent variable. Analysis are adjusted for treatment site (Amsterdam, Arnhem or St Louis). Covariates will be: 1) KAFO wearing time, 2) lower leg growth, 3) use of an AFO by day, 4) stretching exercises and 5) level of spasticity. The α-value will be set at 0.05.

## Discussion

This randomised controlled trial focuses on the efficacy of orthotic management in rest in children with CP to prevent a decline in ankle dorsiflexion at full knee extension. Although orthotic management in rest in children with CP is widely applied, very little is known about the efficacy of this treatment. Therefore, more research on the efficacy of orthotic management in rest is needed. We decided to focus on the efficacy of two types of KAFOs as orthotic management in rest treatment since we expect that KAFOs will be the most effective in preventing loss of ankle dorsiflexion range. This expectation is based on the fact that a KAFO, in contrast to an ankle-foot-orthosis (AFO), is able to impose both knee extension and ankle dorsiflexion simultaneously. This may lead to more stretch on the *Gastrocnemius muscle *which is the target muscle of this study. The *Gastrocnemius muscle *is often affected in children with spastic CP, having equines [9].

To measure the efficacy of orthotic management in rest, the ankle dorsiflexion has to be measured in a reliable and valid way. In order to achieve that, a custom designed hand held dynamometer [31] is used to measure the ankle dorsiflexion angle. The device allows reproducible measurements of the ankle angle-moment relationships [31]. This is important as ankle dorsiflexion depends on the exerted dorsiflexion moment. Standardization of this measurement procedure reduces variability of ankle moment and angle measurements and hence increases the sensitivity to measure changes in ROM. Furthermore, the device allows the investigator to correct for foot deformations by using two separate adjustable foot plates (to stabilize the talonavicular joint) [31]. Foot deformations like equinovarus and equinovalgus [44] can, if not corrected, affect the ankle dorsiflexion measurement results.

The custom designed hand held dynamometer also allows us to measure morphological parameters of the *medial Gastrocnemius muscle *at a standardized ankle position during repeated measurements. By measuring changes in the morphological parameters and changes in ankle ROM simultaneously, we will gain insights into the working mechanisms of orthotic management in rest. As described in the methodological section we obtain the morphological parameters by a 3D ultrasound method. We decided to use this method instead of a 2D ultrasound technique, because 3D ultrasound offers the advantage of measuring the morphological parameters in the mid-longitudinal fascicle plane. Previous research has shown that measuring outside of the mid-longitudinal fascicle plane leads to over or under estimation of the results [43].

The primary outcome measure of this study (ankle dorsiflexion ROM) is an impairment parameters at the body-function level of the International Classification of Function, disability and health (ICF) [45]. Improvements (or less decline) in this parameter do not necessarily lead to improvements in activity level of the ICF model, such as walking [45]). In order to better understand the impact of orthotic management during rest on activity level, this study will also investigate improvements in gait and gross motor function level in response to treatment.

In conclusion, this randomised controlled trial is aimed to provide insight in the efficacy of orthotic management in rest on body function level (ankle dorsiflexion range of motion) and activity level (gait, gross motor function), and will provide insight in the treatment's underlying myological adaptive mechanisms. The results of this study may provide indications for improved treatment strategies for children with cerebral palsy and in particular the use of orthoses.

## Abbreviations

CP: Cerebral Palsy; ROM: Range Of Motion; KAFO: Knee-Ankle-Foot Orthosis; *ℓ*_(fasc)_: Fascicle length; *ℓ*_m_: Muscle belly length; A_f_: Physiological cross- sectional area; *γ_(fasc)_*: Angle between fascicle and aponeurosis; *ℓ*_t_: Tendon length; AFOs: Ankle-Foot Orthoses; *γ_(f-t)_*: Angle between the footplate of the foot fixation and the tibia; EMG: Electromyography; MVC: Maximal Voluntary muscle Contraction; GMFM-66 IS: Electromyography; GMFCS: Gross Motor Function Classification System; AOC: Angle Of Catch; SCALE: Selective Control Assessment of the Lower Extremity; *ℓ*_(m th)_: Muscle thickness; *ℓ*_a_: Length of the aponeuroses; *ℓ*A_f_: Length component of the physiological cross-section; ICF: International Classification of Function, disability and health.

## Competing interests

The authors declare that they have no competing interests. Although Ultraflex Europe made a donation to this study on behalf of Ultraflex Systems Inc, and although PEDAK Meettechniek BV sponsored the "Tidbit temperature data loggers" partly, these companies will not have any authority over this study. Measurements and data-analyses will be performed independently by the researchers.

## Authors' contributions

JCM participated in the design (morphological & clinical part), and drafted the manuscript. JGB, AJD, RTJ and PAH participated in the design (morphological & clinical part). JEB, PJK participated in the design (clinical part). All authors participated in the reviewing process and approved the final manuscript.

## Pre-publication history

The pre-publication history for this paper can be accessed here:

http://www.biomedcentral.com/1471-2431/12/38/prepub
